# Interfacial Behavior of Cubosomes: Combined Langmuir–Blodgett/Langmuir–Schaefer and AFM Investigations

**DOI:** 10.1021/acs.langmuir.3c00810

**Published:** 2023-05-26

**Authors:** Michalina Zaborowska, Aleksandra Bartkowiak, Ewa Nazaruk, Dorota Matyszewska, Renata Bilewicz

**Affiliations:** † Faculty of Chemistry, University of Warsaw, Pasteura 1, 02093 Warsaw, Poland; ‡ Faculty of Chemistry, Biological and Chemical Research Centre, University of Warsaw, Żwirki i Wigury 101, 02089 Warsaw, Poland

## Abstract

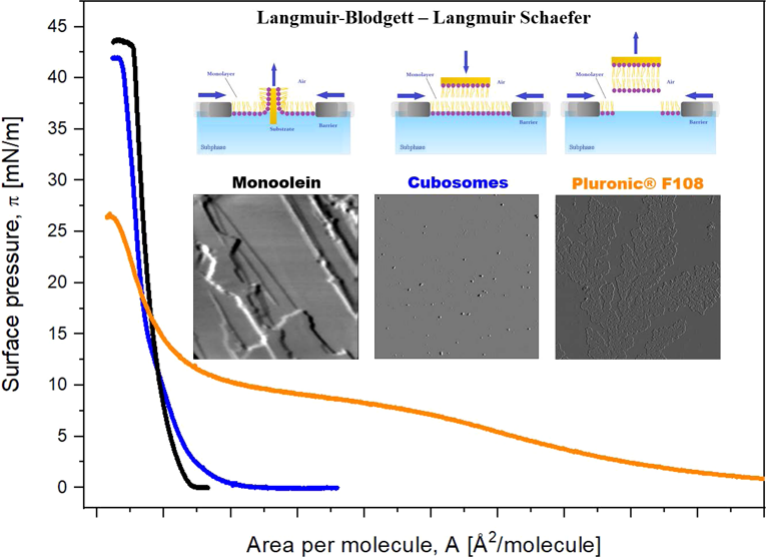

The Langmuir technique was applied for the first time to compare the layers obtained by spreading lipid liquid-crystalline nanoparticles monoolein 1-oleoyl-rac-glycerol (GMO)/Pluronic F108 cubosomes with the monolayers obtained by mixing the same components in chloroform at the air–water interface. The differences in the monolayer behavior and in the acting intermolecular forces were examined. The similarity of the isotherms obtained for the mixed components system and the cubosome-derived layer proved the disintegration of cubosomes into a single monolayer upon contact with the air–water interface. Despite the low Pluronic F108 content in both types of layers, a strong structural role of this stabilizer was also demonstrated. Cubosome-derived systems supported on hydrophilic mica substrates were prepared either using the combined Langmuir–Blodgett and Langmuir–Schaefer technique or via direct adsorption from the solution. The topographies of the obtained layers were studied by atomic force microscopy (AFM). Images obtained in the air mode revealed the disintegration of cubosomes and the formation of large crystallized structures of the polymer, while AFM imaging performed in water confirmed the presence of intact cubosomes on the surface of mica. We proved that the original structure of cubosomes remains on one condition: the films must not dry out; therefore, the aqueous environment must be preserved. This new approach provides an explanation in the ongoing discussion of what happens to lipid nanoparticles with or without cargo when they come into contact with an interface.

## Introduction

Lipid liquid-crystalline phases and their nanoparticles, such as cubosomes, are highly adaptable and promising drug carriers. They comprise a cubic phase of lipids with a polymer as a stabilizing agent. Biocontinuous lipid cubic phase is a continuous periodic structure of a lipid membrane in which water channels are present.^1−3^ One of their biggest advantages is the possibility of accommodating hydrophobic and hydrophilic molecules and a regulated release of these individuals. The cubosome structure provides a large active surface area for loading membrane proteins and drug molecules. In addition, cubosomes have the potential to deliver antimicrobials,^4^ anticancer agents,^5^ imaging contrast agents,^6^ and bioactive lipids.^7,8^ As nanoreactors or biosensors, cubosome systems have exciting applications.^9^

Individual nanocarriers must interact with the cell surface for drug delivery via nanocarriers. This is the first obstacle they encounter while delivering drugs to the target. Research described in the literature focuses on the adsorption of cubosomes to the surface of cells, where lipid exchange between cubosomes and cell membrane can take place, as well as endocytosis as a potential cubosome–cell interaction. Few studies have described the molecular interactions between cubosomes and model biological membranes.^10−15^ According to Vandoolaeghe et al.,^10^ the interaction between cubic nanoparticles and the lipid bilayer is a dynamic process, which consists of the initial adoption of the nanoparticles on the surface of the bilayer. Shen et al.^11^ found that cubosomes do not adhere directly to the silica-supporting surface.

Recently, our team studied the interactions between phytantriol-based cubosomes and model biological membranes generated via the Langmuir–Schaefer (LS) and Langmuir–Blodgett (LB) methods. We have shown that cubosomes expand upon contact with the 1,2-dipalmitoyl-*sn*-glycero-3-phosphocholine Langmuir monolayer and that the molecular packing of the monolayer, which determines the free adsorption sites, determines also the transfer of GMO molecules from the cubosomes into the lipid layer.^16^ With more porous lipid layers, the disintegration action of cubosomes is more intense, and drug delivery can be more efficient. In contrast, extremely densely packed compact monolayers were impermeable to cubosomes; hence, they remained below the monolayer which covered the air–water interface.

Regarding the potential use of cubosomes as a technological platform for the delivery of active agents through interaction with surfaces,^17^ it is crucial to understand the surface characteristics when it is exposed to cubosomes and the mechanism of cubosomes disintegration at the air–water interface.

**Figure 1:**
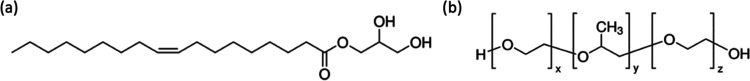
Chemical structures of (a) monoolein (GMO) and (b) polymer Pluronic F108.

In this study, for the first time, we compare the behavior of GMO/Pluronic F108-based cubosomes and their individual components (Figure 1) at the air–water interface and the results of modifying solid substrates (mica) with these compounds. The decomposition of the cubosomes on the subphase of water into a mixed monolayer is demonstrated and a thermodynamic characterization of the tested systems is included. Using atomic force microscopy (AFM), the morphological properties of cubosomes dispersed over a mica support were investigated. The observed data are expected to aid in better understanding of the cubosome interactions with surfaces.

## Experimental Section

### Materials

GMO, Pluronic F108, and chloroform used to synthesize the mesophases were purchased from Merck. Ultrapure water (MilliQ) (18.2 MΩ cm^–1^; Millipore) was used in the experiments.

### Cubosome Preparation and Characterization

Using a top-down procedure, cubosomes were prepared from GMO and the polymer Pluronic F108. A GMO sample was hydrated with water and the Pluronic F108 stabilizer (lipid:Pluronic F108 molar ratio was 0.996:0.004). The sample was then homogenized for 20 min using SONICS Vibracell VCX 130 (Sonics & Materials Inc.) at an amplitude of 40% (2 s pulses and 3 s breaks). The generated cubosomal dispersions were structurally characterized by small-angle X-ray scattering (SAXS), and the phase identity of the mesophases was obtained with a Bruker Nanostar system equipped with a Vantec 2000 area detector (Cu Kα radiation was used). Samples were injected into 1.5 mm capillaries and measured at room temperature. Before measurements, cubosomes were allowed to equilibrate at room temperature overnight. Upon exposure to X-rays, diffraction rings were observed, which were then applied to differentiate the mesophase. The 2D patterns were integrated to yield intensity, *I*(*q*), vs scattering vector, *q*, 1D plot, where *q* = (4π/λ)sin(θ), λ = 1.54 Å, and 2θ is the angle between the incident and scattered X-rays. The ratios of *q* values for the peaks were used to determine the Miller indices for each peak to reveal the identity of the mesophase.

In the cryo-TEM experiment, 3 μL of the sample was deposited onto the glow discharged holey carbon grids (Quantifoil R2/1) using Vitrobot MarkIV (Thermo Fisher). 2D electron cryo microscopy images were taken on a Glacios TEM (Thermo Fisher) operating at 200 kV. Images were captured on a Falcon 3EC direct electron camera at a magnification of 92 k.

Dynamic light scattering experiments were made using a Zetasizer Nano ZSP (Malvern Panalytical, Malvern, U.K.). The sizes of the cubosomes were measured in quartz cuvettes with MilliQ water (25 °C). The cubosome solution was diluted 50-fold in MilliQ water. The size distribution of the nanoparticles was expressed as the hydrodynamic diameter distribution.^18^ The analysis was performed using the specialized Malvern software.

### Langmuir Technique and Isotherm Analysis

Langmuir experiments were conducted by means of a KSV Nima Langmuir trough (Biolin Scientific, Sweden) of the total area of 243 cm^2^, which was equipped with two hydrophilic barriers and a computer-controlled software. A Wilhelmy plate made of filter paper served as a surface pressure sensor. Prior to each experiment, the trough was cleaned with methanol and chloroform and thoroughly rinsed with MilliQ water. GMO and Pluronic F108 solutions in chloroform had the final concentration of 1 mg/mL. The mixed GMO/Pluronic F108 solution with the concentration of 1 μmol/mL and the 0.996:0.004 molar ratio corresponding to the composition of the cubosome formulation was prepared by mixing the appropriate volumes of individual components’ solutions. After spreading the GMO, Pluronic F108, mixed GMO/Pluronic F108, or cubosome solution (diluted approx. 50 times in MilliQ water) on the pure water subphase, the film was symmetrically compressed at a constant rate of 10 mm/min (7.5 cm^2^/min) while concurrently recording the surface pressure (π)–area per molecule (*A*) isotherm. All of the experiments were conducted at room temperature (21 ± 1 °C).

Using the π–*A* isotherm data, the following parameters were determined: the lift-off area (*A*_lift-off_), which is the region where the transition from the gas phase to the liquid-expanded phase takes place, limiting area (*A*_0_), which is the area per lipid molecule reflecting a well-organized monolayer, and max*C*_S_^–1^, which is the maximum compression modulus value. The compression modulus (*C*_S_^–1^) values were calculated according to the following formula:^19^
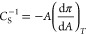
1

This modulus (eq 1) provides information regarding the physical state of the film, its elasticity, and the ordering of the molecules during compression. The *C*_S_^–1^ values range from 12.5 to 100 mN/m for liquid-expanded (LE) films, from 100 to 250 mN/m for liquid-condensed (LC) films, and above 250 mN/m for solid films (*S*).^20^

Directly from the π–*A* isotherms, the mean area per molecule in the mixed binary film (*A*_12_) at different surface pressures may be calculated and compared to the value corresponding to ideal miscibility or complete lack of particle miscibility (eq 2):^21,22^
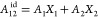
2where *A*_1_ and *A*_2_ are the molecular areas of single components 1 and 2, while *X*_1_ and *X*_2_ correspond to the mole fractions of these components in the mixed film.

The excess area per molecule (*A*^Exc^) in the mixed binary monolayer was determined according to the following equation:
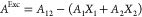
3where *A*_12_ is the area per molecule value of the multicomponent layer under a given surface pressure.

For the cases of hysteresis occurring during the cyclic compression and expansion of the Langmuir monolayer, the free energy of hysteresis (Δ*G*_hys_), the configurational entropy of hysteresis (Δ*S*_hys_), and the enthalpy of hysteresis (Δ*H*_hys_) were determined using the following equations:^23−25^
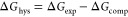
4
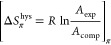
5
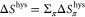
6
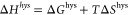
7

The free energy of compression (Δ*G*_comp_) and the free energy of expansion (Δ*G*_exp_) were calculated using , where *N* is the Avogadro number, and the integral is calculated between π_1_ = 1 mN/m and π_2_ = 40 mN/m for both the compression and expansion cycles. With perfectly fluid films, hysteresis does not exist; hence, the thermodynamic functions of hysteresis are 0. However, in real systems with hysteresis present, the deviations of the values of thermodynamic functions of hysteresis from 0 are observed. Moreover, the information on the energy trapped due to the cohesive intra- and intermolecular forces in the monolayer can be extracted from the change in free energy during compression and expansion (Δ*G*_comp_ and Δ*G*_exp_).^24,25^

### Solid Surface Modification

#### LB–LS Deposition

The two-step process for obtaining the bilayers involved the transfer of the first leaflet onto a solid substrate via the LB method, i.e., vertical lifting of the mica substrate, previously immersed in the water subphase, through the monolayer-covered air–water interface. The monolayer was transferred to mica under continuous surface pressure of 20 mN/m. The barrier speed was 10 mm/min during compression, whereas the substrate withdrawal speed was 15 mm/min. The modified surface was allowed to dry for 60 min. Next, the second leaflet was transferred via the LS method, i.e., the horizontal touch procedure. Under the same experimental conditions, LS films were transferred by touching the LB film-covered substrate horizontally to the compressed LS film and slowly (0.5 mm/min) elevating it upward. After this procedure, AFM imaging was conducted.

#### Adsorption from Solution

Adsorption from the solution, involving the self-assembly of cubosomes forming the layer-like structure on hydrophilic mica, is the second way to obtain layers on a solid support.^26,27^ Cubosomes are hydrophilic on the outside and their adsorption to the surface is a simple process. Two hundred microliters of a cubosome solution (concentration: 0.4 mg/mL) was applied to the freshly sliced mica surface and left for 4 h for the adsorption process.^28^ After that, the remaining solution was rinsed with MilliQ water for the specified duration and mica plates were allowed to dry (30 min). These samples were used to study the behavior of cubosome layers at the air–mica interface. In addition, the samples generated in this way (adsorption time: 4 h) were tested in MilliQ water. After 4 h of adsorption, the plate was rinsed to remove excess cubosome solution and submerged in MilliQ water.^29^ The adsorption process of cubosomes from an aqueous solution was also monitored with time to determine when the structures on the mica surface became highly stable. In this case, MilliQ water was dripped onto the mica surface; then, the cubosome solution was injected to reach a final concentration of 0.4 mg/mL.

### Atomic Force Microscopy Imaging

#### Preparation of the Mica Surface

Mica plates with a diameter of 20 mm were used as substrates for layer transfer using the LB–LS and adsorption methods. The plate was washed with chloroform to prepare the substrate, and its thin layer was peeled off using adhesive tape.^30,31^ The contact angle of approximately 35° demonstrated that clean mica plates were hydrophilic.

AFM measurements were performed using Dimension Icon (Bruker, Billerica, MA, USA) in Peak Force QNM mode to characterize the coated mica in terms of its topography and film thickness. The *z*-piezo frequency was modulated at 2 kHz. ScanAsyst Fluid+ and probes with an elasticity constant of *K* = 0.7 N/m were used. Due to the characteristics of the soft particles, very slow scanning of large surfaces is recommended.^27^ After processing the images, we attempted to flatten the background to visualize the layers generated by cubosomes using Gwyddion software.^31^ In order to determine the average layer thickness, height distribution histograms were plotted by collecting heights from at least three different cuts.

## Results and Discussion

### Characteristics of Cubosomes

**Figure 2:**
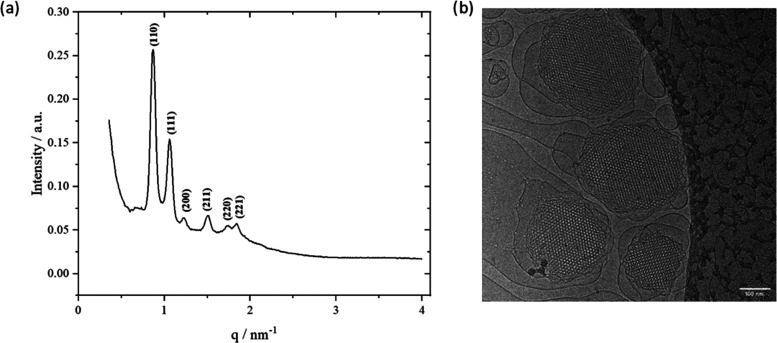
(a) Representative small-angle X-ray scattering diffraction patterns obtained for (GMO)/Pluronic F108-based cubosomes. The observable Bragg peaks are indexed with the corresponding Miller indices (hkl). The Bragg peaks correspond to the [110], [111], [200], [211], [220], and [221] reflections of a double diamond *Pn*3̅*m* cubic phase. (b) Cryo-TEM image visualizing the GMO/Pluronic F108 cubosome sample.

SAXS analysis (Figure 2) confirmed the presence of a double diamond cubic phase (*Pn*3̅*m*) in the (GMO)/Pluronic F108 formulation. At 25 °C, the SAXS profile exhibits a sequence of diffraction peaks with relative positions at ratios of √2:√3:√4:√6:√8:√9, which can be attributed to the double diamond (*Pn*3̅*m*) cubic symmetry. The lattice parameter (*a*) of the cubosomes was 10.3 nm.

**Figure 3:**
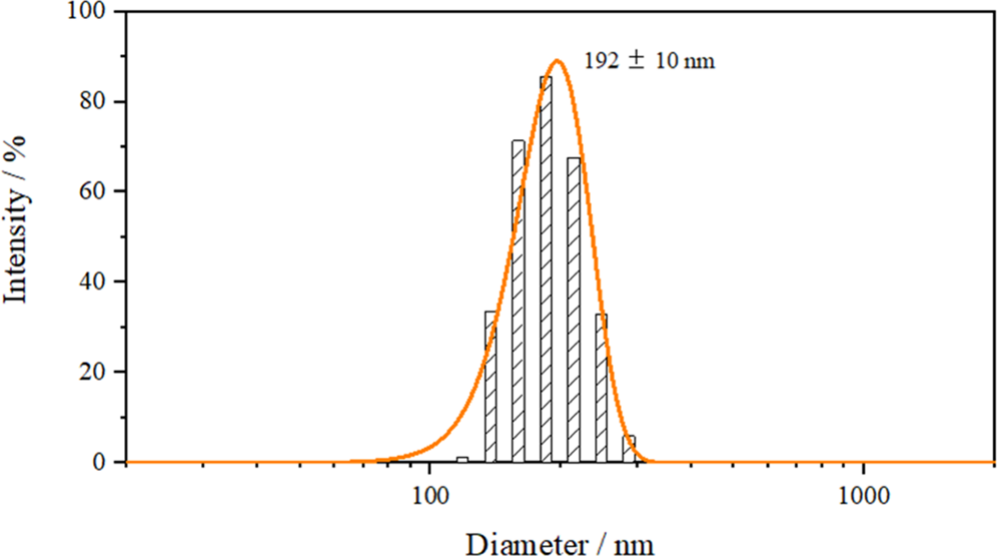
Calculated Gaussian distributions of the particle sizes determined using dynamic light scattering for the cubosomes (orange).

The DLS method uses the hydrodynamic diameters of cubosome structures to determine their sizes. The histogram indicated a mean diameter of 192 nm (Figure 3), which is also confirmed by Cryo-TEM images (Figure 2b). Moreover, the size distribution was relatively homogeneous. The cubosomes presented here are comparable in size to those reported in the literature for cubosomes composed of GMO (94%) and Poloxamer (6%).^27^

### Behavior of Cubosomes at the Air–Water Interface

**Figure 4:**
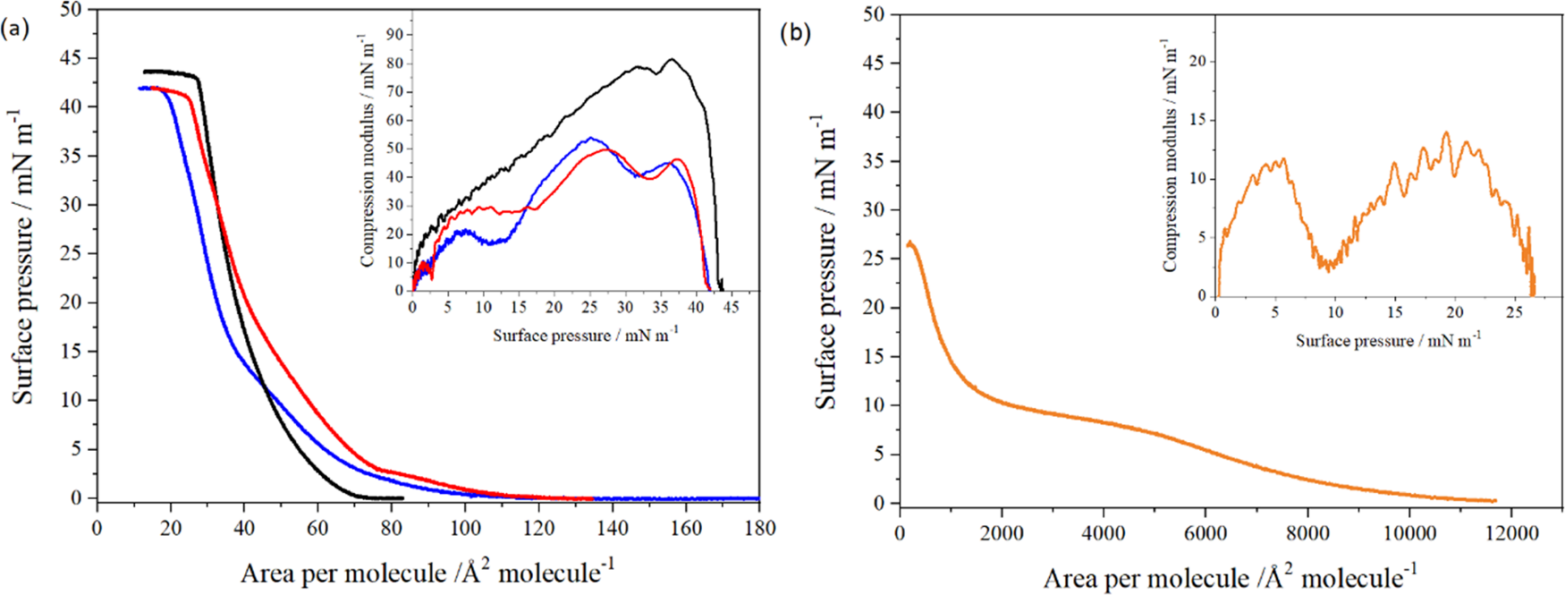
Surface pressure (π)–area per molecule (*A*) isotherms of a Langmuir monolayer consisting of (a) GMO (black), the mixture of GMO/Pluronic F108 in chloroform (red), and GMO/Pluronic F108 cubosome formulation with the same ratio of components (blue) and (b) a single-component Pluronic F108 monolayer. All isotherms were recorded in the ultrapure water subphase. Inset: the plot of compression modulus versus surface pressure.

In the first part of the work, Langmuir experiments were conducted to compare the interfacial properties of GMO-based cubosomes and their components mixed in the same ratio. Figure 4 shows the π–*A* isotherms recorded for monolayers generated using GMO, Pluronic F108, GMO/Pluronic F108 in chloroform, and a cubosome formulation dispersed on an ultrapure water subphase. The maximum values of *C*_S_^–1^ indicate that all of the monolayers are in the LE phase (Table 1). The π–*A* isotherm obtained for a monolayer composed of mixed GMO/Pluronic F108 retraces the isotherm obtained for a monolayer generated by placing a cubosome sample at the interface (Figure 4). In addition, two local minima were observed on each isotherm at surface pressures of approximately 15 mN/m and approximately 33 mN/m (Figure 4). The minimum observed at lower surface pressure corresponds to the minimum occurring for pure Pluronic F108 monolayers but at a somewhat lower surface pressure of approximately 10 mN/m. The second minimum of the compression modulus, observed at approximately 33 mN/m, corresponds to the removal of Pluronic F108 from monolayers composed of the GMO/Pluronic F108 mixture and from the cubosome formulation.

**Table 1:** Parameters of the π–*A* Isotherms for Langmuir Monolayers Formed Using Monoolein 1-Oleoyl-rac-glycerol (GMO), Pluronic F108, GMO/Pluronic F108, and GMO/Pluronic F108 Cubosome Formulation

monolayer	*A*_lift-off_ [Å^2^]	*A*_0_ [Å^2^]	max*C*_S_^–1^ [mN/m]
GMO	71.0 ± 1.8	44.5 ± 1.4	81.0 ± 2.3
GMO/Pluronic F108	108.5 ± 2.5	55.2 ± 1.5	49.7 ± 1.5
GMO/Pluronic F108 cubosome formulation	115.0 ± 2.0	46.0 ± 1.1	54.2 ± 2.0

**Figure 5:**
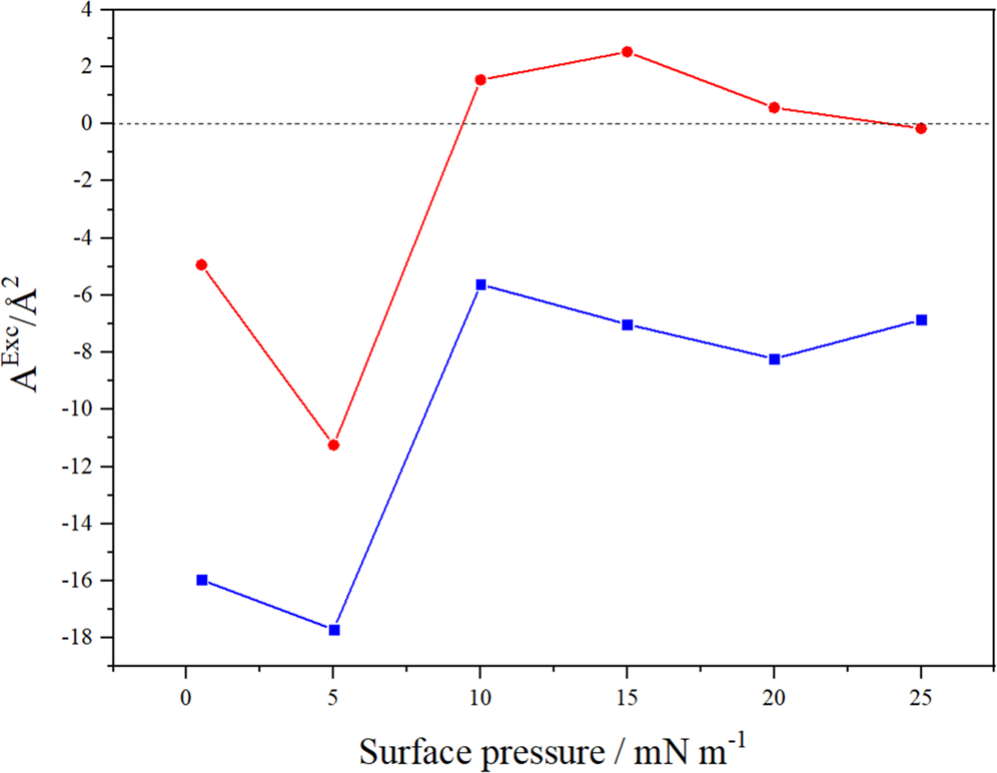
Values of the excess area (*A*^Exc^) calculated at selected surface pressures for GMO/Pluronic F108 in chloroform (red/●) and the cubosome-derived layer (blue/■).

The miscibility of the components in the studied monolayers can be inferred by analyzing the values of *A*^Exc^.^19,32^ As shown in Figure 5 (red), large negative values of the excess area at low surface pressures (π < 10 mN/m) indicate good miscibility of monolayer-forming GMO and Pluronic F108 molecules. In turn, further compressing the monolayer to higher surface pressures diminishes the miscibility of the components. Nevertheless, components in the cubosome-derived layer are considerably more miscible, as demonstrated by large negative values across the entire range of surface pressure values (Figure 5 blue).

**Figure 6:**
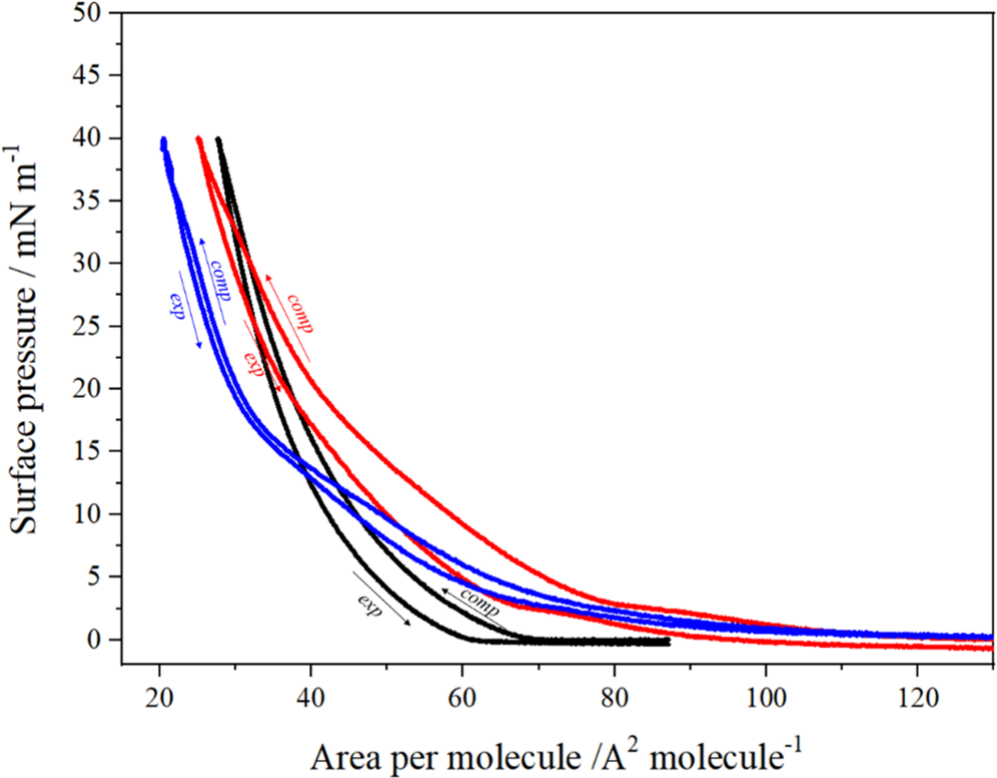
Compression–expansion cycles for GMO (black), the mixture of GMO/Pluronic F108 in chloroform (red), and cubosome formulation spread on the subphase (blue) and compressed to 40 mN/m on ultrapure water.

In addition, the recorded compression–expansion cycles (Figure 6) allowed for the assessment of thermodynamic properties, energy effects, and the possibility of aggregate formation in the studied monolayers composed either of a mixture of GMO and Pluronic F108 or the cubosome-derived layer on the subphase surface.

The hysteresis experiment illustrated in Figure 6 demonstrates that the mean molecular areas of GMO and GMO/Pluronic F108 are shifted to smaller values in the decompression half-cycle. Interestingly, for cubosome-derived layers, the hysteresis is smallest. The presence of hysteresis indicates that the mixed monolayer structure is disturbed due to the removal/aggregation of the polymer component.

Additional information was provided by the calculated thermodynamic parameters of the hysteresis: Δ*G*^hys^, *T*Δ*S*^hys^, and Δ*H*^hys^ (Table 2). For ideally elastic layers without the formation of any irreversible aggregates, the values of the thermodynamic parameters should be equal to zero. In the case of the GMO monolayer, GMO/Pluronic F108, and cubosome formulation, the Δ*G*^hys^ values are slightly negative, which indicates a limited accumulation of free energy in the cycle. The magnitude of the observed changes provides quantitative information on the free energy trapped due to the presence of kinetically limited viscoelastic effects as well as the cohesive inter- and intramolecular interactions in the monolayer. Additionally, both *T*Δ*S*^hys^ and Δ*H*^hys^ values are negative, indicating entropically unfavorable layer organization and, consequently, enthalpically favorable interactions during compression.^24^ These values vary substantially in the case of a mixed monolayer (GMO/Pluronic F108) (Table 2), indicating that the presence of Pluronic F108 affects the thermodynamic parameters of a monolayer composed of GMO molecules and leads to a more compact and ordered molecular organizations in the mixed monolayer. In contrast, the values of three parameters characteristic of hysteresis recorded after the spreading of cubosomes at the air–water interface suggest more favorable interactions between GMO and Pluronic F108 than in the case of simple mixing of these two components. In the latter case, aggregates may form at the air–water interface and do not easily disperse during the membrane expansion cycle in contrast to the layer formed by the cubosomes.

**Table 2:** Thermodynamic Functions Calculated from the Hysteresis Experiments: the Free Energy of Compression (Δ*G*_comp_), the Free Energy of Expansion (Δ*G*_exp_), the Difference (Δ*G*^hys^), the Configurational Entropy of Hysteresis (*T*Δ*S*^hys^), and the Enthalpy of Hysteresis (Δ*H*^hys^) Calculated between π = 1 mN/m and π = 40 mN/m for Monoolein 1-Oleoyl-rac-glycerol (GMO) and GMO/Pluronic F108 Langmuir Monolayer Formed in Ultrapure Water Subphase

	Δ*G*_comp_ [kcal mol^–1^]	Δ*G*_exp_ [kcal mol^–1^]	Δ*G*^hys^ [kcal mol^–1^]	*T*Δ*S*^hy*s*^ [kcal mol^–1^]	Δ*H*^hys^ [kcal mol^–1^]
GMO	0.71 ± 0.01	0.54 ± 0.0	–0.17 ± 0.0	–1.39 ± 0.30	–1.56 ± 0.30
GMO/Pluronic F108	1.28 ± 0.05	0.92 ± 0.08	–0.36 ± 0.02	–2.45 ± 0.40	–2.81 ± 0.40
GMO/Pluronic F108 cubosome formulation	1.01 ± 0.04	0.89 ± 0.07	–0.12 ± 0.02	–0.99 ± 0.30	–1.11 ± 0.35

### Topography of the Single-Component GMO and Pluronic F108 Films Transferred from the Air–Water Interface onto Gold Electrode

The GMO monolayer was transferred from the Langmuir trough to the mica substrate at the surface pressure of 20 mN/m using the LB–LS method. As demonstrated by the Langmuir measurements (Figure 4), GMO forms liquid layers; therefore, measurements at lower surface pressure levels were excluded. The LB–LS method yields GMO films with multilayer properties comprising bilayers (Figure S1a,b), there is no difference between the topography of the layers registered in air and in solution (MilliQ water). At 20 mN/m, the histograms show an average height of 8 nm, indicating the presence of at least two bilayers. Interestingly, the LB–LS layer transfer of Pluronic F108 (Figure S2) resulted in the formation of nonhomogeneous nanostructures when the plates were left to dry and measured in air.^33^ Heterogeneous large domains (5 μm × 5 μm) embedded in the bilayer matrix with an average height of 6 nm relative to the bilayer (4 nm) can be noticed. Measurements carried out in the solution revealed no formation of domains characteristic to the polymer and quite homogeneous layers with a thickness of 2 nm (with small build-ups on the surface) were obtained. It can therefore be concluded that the formation of the above-mentioned nanostructures is caused by the drying process.

### Topography of the Mixed GMO/Pluronic F108 and GMO/Pluronic F108 Cubosome-Derived Films Transferred from the Air–Water Interface

**Figure 7:**
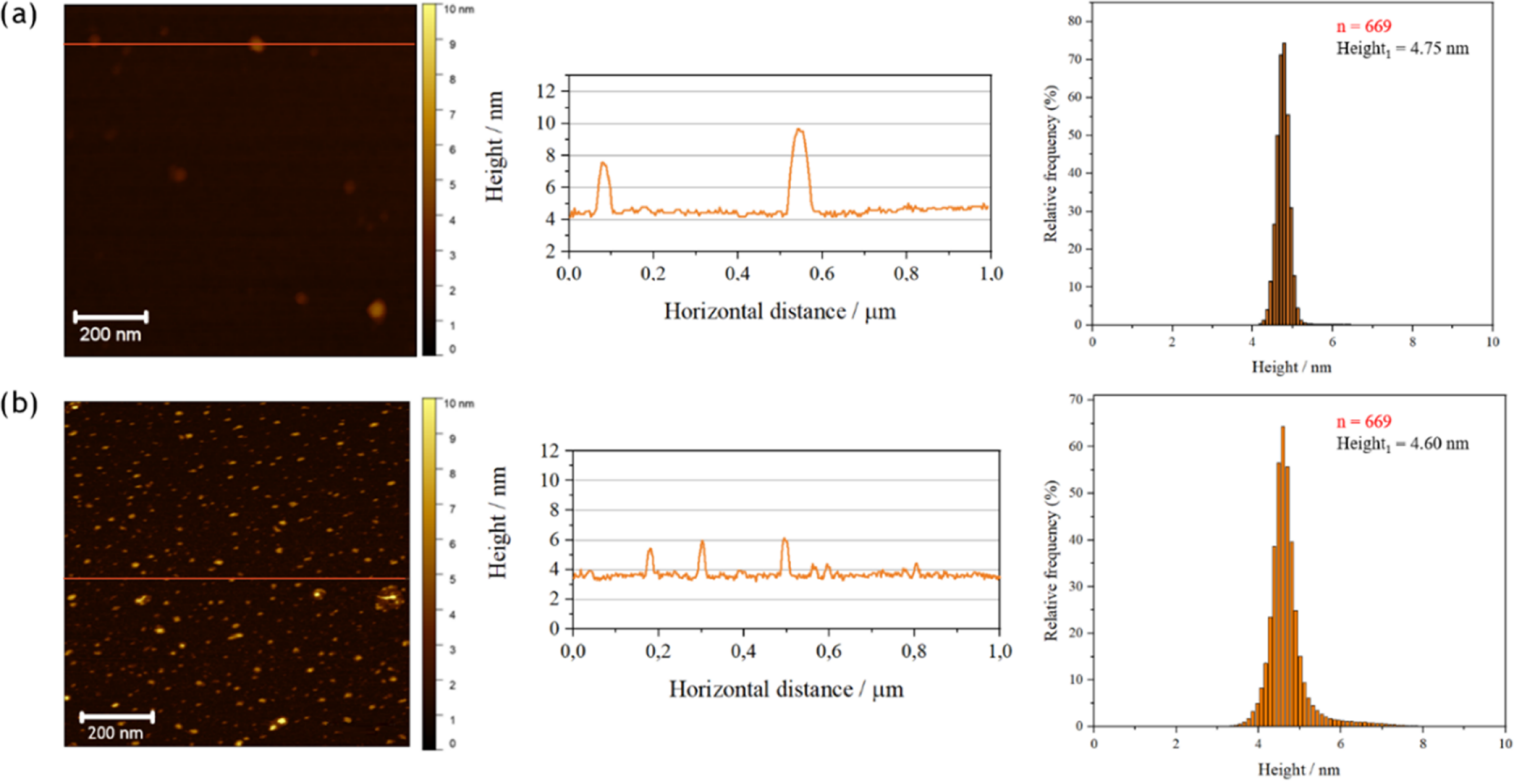
AFM images of the mixed GMO/Pluronic F108 layers obtained using the LB–LS method at the surface pressure of 20 mN/m (a) in air and (b) in water (left column). The orange lines correspond to the profiles (middle column). Average height distribution histograms are collected for the selected areas (1 μm × 1 μm, red squares shown in Figure S3) of the obtained images (right column).

Figure 7 compares the topography of the mixed GMO/Pluronic F108 layers with that of films of GMO/Pluronic F108 dispersed cubosomes transferred onto a mica substrate and measured in two modes: in air and solution.

Similarly to single-component layers, the mixed GMO/Pluronic F108 layers transferred through the LB method did not adhere to the mica surface (data not shown). In the LB–LS approach, the layers adhered better and were more stable (Figures 7 and S3). According to Langmuir investigations, the polymer is progressively removed from the mixed GMO/Pluronic F108 monolayer at the surface pressure above approximately 30 mN/m (Figure 4). Therefore, the mixed layers were transferred at the lower surface pressure of 20 mN/m, ensuring the presence of the polymer in the transferred layer. The image obtained in the air confirms a well-organized, somewhat homogeneous bilayer. The histograms compiled from many patterns (1 μm × 1 μm) extracted from the larger image in Figure S3 prove that the thickness of the layer is approximately 4.7 nm, which is comparable to the mean thickness of the GMO bilayer (Figure S1). However, the interactions between the GMO and the Pluronic F108 polymer occurring in the mixed layer prevent the formation of multilayers on top of the bilayer, which was observed for the GMO film alone. In the case of the measurements in the water, the general characteristics are similar; the average thickness of the layer is also about 4.7 nm (Figure 7b). The larger circular structures can indicate Pluronic F108 domains. It is supported by the fact that at a surface pressure higher than 10 mN/m, the excess area becomes positive (Figure 5), which suggests that the forces between the components in the mixed layer are more repulsive or less attractive than in the single-component layers. Thus, the phase separation in the layer can be observed, which finally leads to the expulsion of the polymer from the layer. Interestingly, when preserving the aquatic environment, the separation of polymer structures is much more visible, although the size of the circular structures seems to be smaller. In the air mode, the structures ascribed to the polymer are fewer but larger (Figure 7a). It might be explained by the fact that layers of Pluronic F108 formed nonhomogeneous nanostructures when left to dry (Figure S2). This process is obviously not observed for the mixed GMO/Pluronic F108 layers but may lead to the formation of larger structures made up of polymer upon drying of the film.

**Figure 8:**
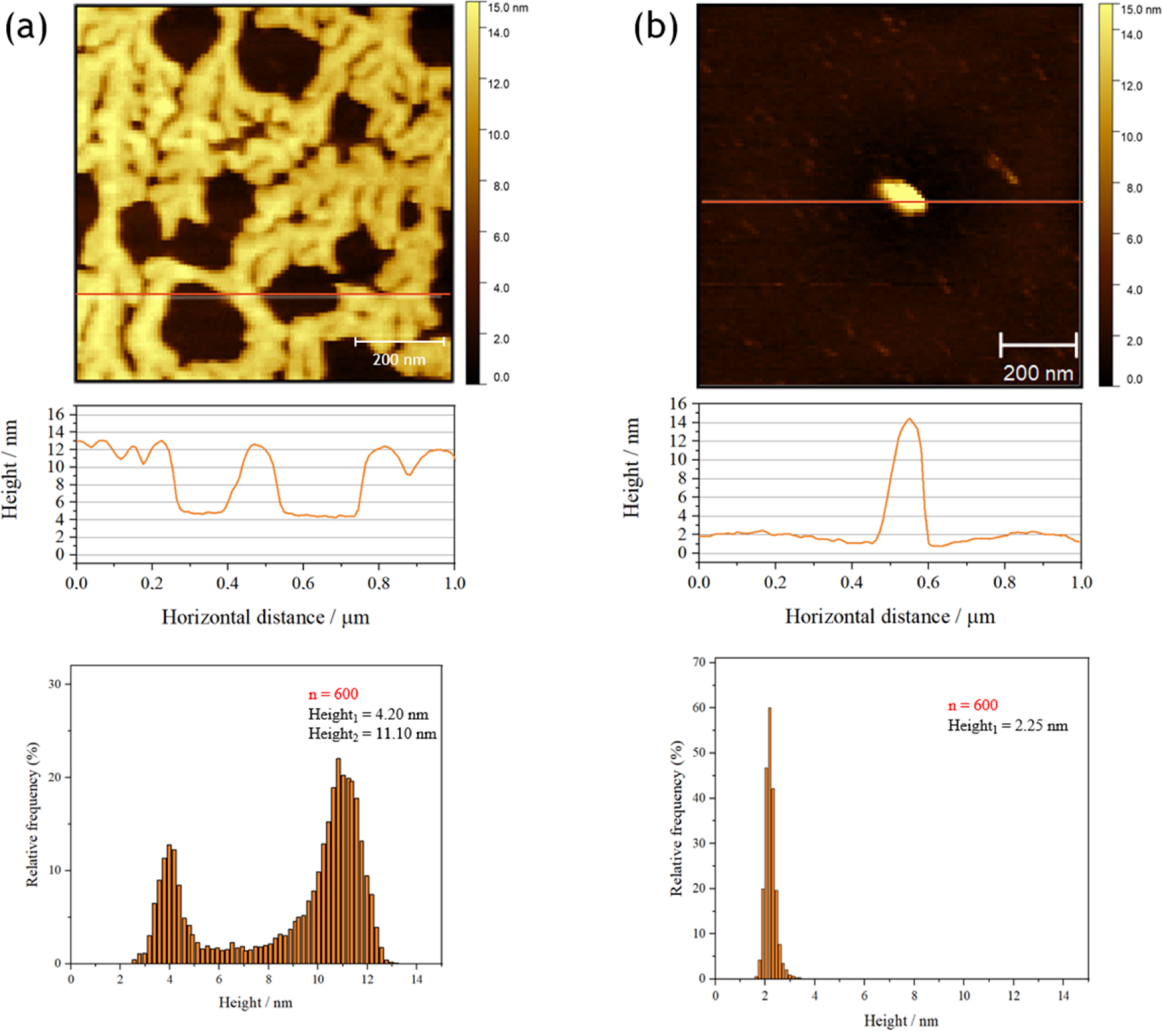
Topographic AFM images of the cubosome-derived layers obtained using the LB–LS method at a surface pressure of 20 mN/m recorded (a) in the air and (b) in the water (upper panel). The orange lines correspond to the profiles (middle panel). Average height distribution histograms collected in the selected areas (1 μm × 1 μm, red squares shown in Figure S4) of the obtained images (lower panel).

Transferring the LB–LS layer generated with GMO/Pluronic F108 cubosomes at a surface pressure of 20 mN/m and imaging in the air leads to the formation of characteristic structures (Figure 8a). On a somewhat flat matrix, large coniferous heterogeneous domains are visible (Figure S4). When analyzing the 20 μm × 20 μm pattern (Figure S4), a 4 nm thick bilayer was observed, with irregular domains of height of about 8 nm and size of 5–10 μm. Despite the above-postulated hypothesis that cubosomes, which are predominantly water-based structures, disperse at the air–water interface into monolayers of the characteristics comparable to the mixed GMO/Pluronic F108 monolayers, the topography of the GMO/Pluronic F108 cubosome layers transferred to mica support by LB–LS does not resemble the topography of the mixed GMO/Pluronic F108 transferred layer (Figure 7a). Comparable structures could be resolved in the LB–LS layers of the Pluronic F108 polymer alone (Figure S2a), indicating that the polymer is primarily responsible for the characteristics of the cubosome-derived layers imaged in the air. Additionally, the observed 4 nm layer may be ascribed to the GMO component of the spread cubosomes, on top of which the polymer formed these characteristic coniferous structures (Figure 8a). As it was in the case of the polymer itself, the stage of drying the layer and its measurement in the air mode has a serious impact on the organization of the layers and both the phase separation and crystallization of Pluronic F108 can be clearly recognized.

If the cubosome-derived layers are not allowed to dry following the LB–LS transfer, the AFM image is entirely different. The topography of the cubosome-derived layer in water resembles better the mixed GMO/Pluronic F108 layers. In larger areas (Figure S4b), from which smaller histograms of the relative frequency of the occurrence of a given height were selected for plotting, a relatively flat, homogeneous layer with a height of approximately 2 nm with spherical build-ups was obtained. The build-ups may be attributed to the individual cubosomes diffusing to the monolayer-covered air–water interface during the Langmuir–Schaefer horizontal touch transfer of the second layer. It is proposed that these cubosomes have not disintegrated into monolayers and retain their intact structure on the first monolayer present already on mica and oriented with the nonpolar headgroups toward the cubosomes. Figure 8b shows the cubosome structure extracted from the larger area of 5 × 5 μm or 20 × 20 μm (Figure S4b). Their height is 14–16 nm and therefore is an order of magnitude smaller than the diameter of 140–180 nm determined by DLS for cubosomes in solution (Figure 3). However, it has to be kept in mind that in the AFM experiment, solid-supported cubosomes are imaged, which is the reason for their flattened shape (adsorption on the mica plates). On the other hand, the flattening of the discussed structures may result from the underestimation of the height of the tip above the substrate surface, which results from the specificity of the measurement in the noncontact mode. These 3D structures are even better recognized when the imaging is performed in the Peak Force Error mode (Figure S4b, gray pattern).

### Characteristics of GMO/Pluronic F108 Cubosomes Adsorbed on Mica from Aqueous Solutions

**Figure 9:**
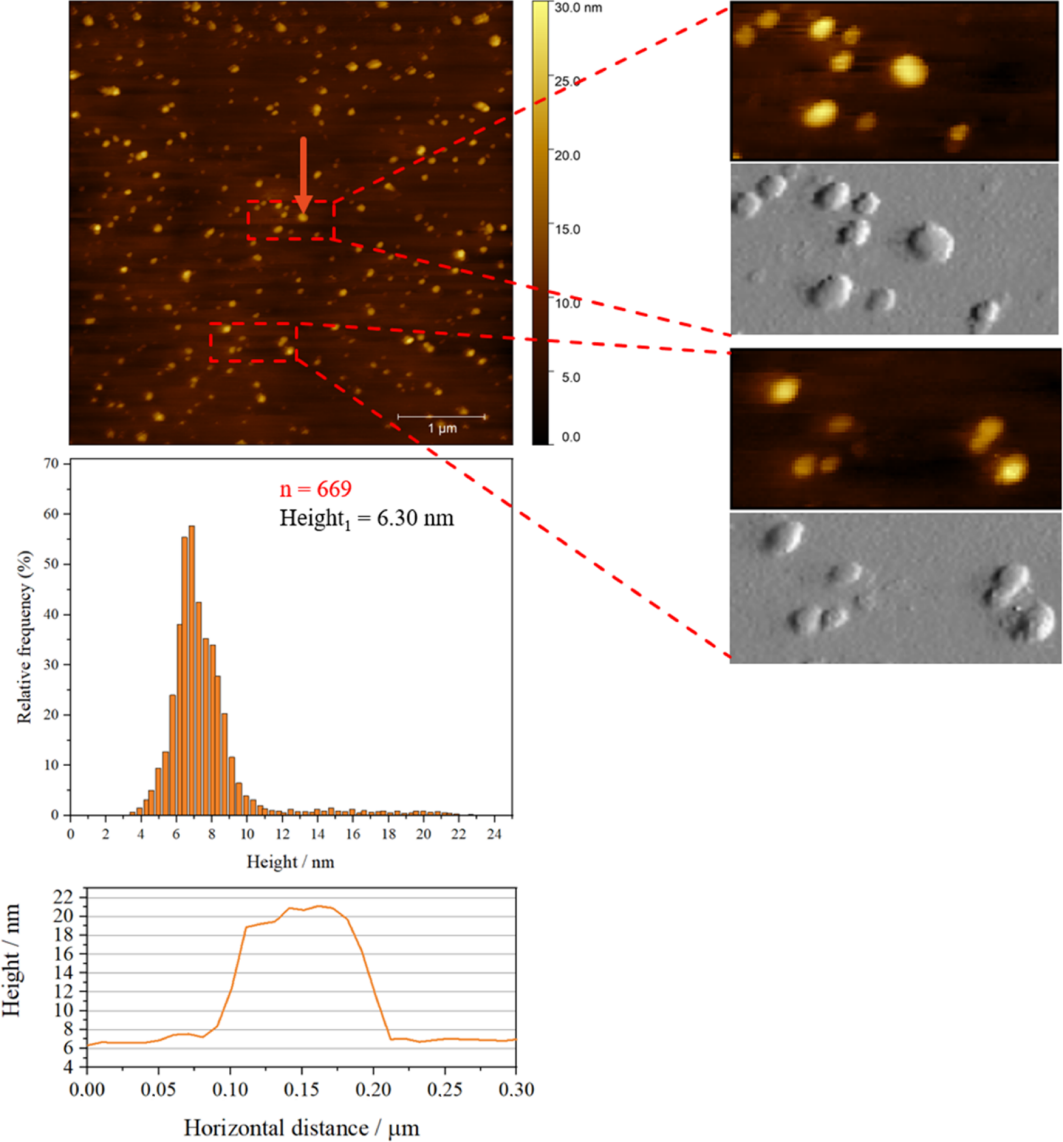
Topographic AFM images of the cubosome layers obtained by adsorbing the cubosomes from the solution onto the mica surface for 4 h and imaging it in water. The orange line and arrow correspond to the profile and arrow. The average height distributions of histograms were collected in the selected areas (5 μm × 5 μm, red squares shown in Figure S5) of the obtained images. The gray patterns are performed in the Peak Force Error mode.

Since a water environment is necessary to retain the structure of a cubosome, in the second approach the cubosomes were simply adsorbed at the mica plates for 4 h. Figure 9 presents the topography of the sample in the aqueous environment.

The images of the cubosomes in water reveal a spherical shape. Their sizes (160–250 nm) are similar to the DLS and cryo-TEM data (Figures 2b and 3). Here, surface adsorption flattens them to approximately 15–20 nm (Figure 9). These results are in-line with the observations of the LB–LS cubosome-derived layers imaged in the water environment and confirm that in order to retain the original nanoparticulate structure, particular care has to be taken to maintain a constant aquatic environment.

## Conclusions

Cubosomes as inverse bicontinuous cubic phase (*Q*_II_) nanoparticles have intriguing features for use as carriers of hydrophilic and hydrophobic molecules, e.g., proteins and drugs. In this paper, we present a comprehensive approach to characterize the cubosomes and the layers they form both at the air–water and solution–solid substrate interfaces. Characterization via SAXS and DLS of the nanoparticles in the bulk solution confirms their double diamond cubic phase structure and the typical diameter of 192 nm.

The Langmuir technique was applied to investigate the behavior of cubosomes at the air–water interface and compare it with the results obtained for single components of GMO and Pluronic F108 as well as their chloroform mixture with the same composition as the one used to prepare cubosomes. The similarity of the shape of the isotherms obtained for the mixture of components placed at the air–water interface and the layer prepared by spreading the original cubosomes suggests that upon contact with the interface, the cubosomes disintegrate into a very similar mixed monolayer. The acquired thermodynamic data indicates some memory effect, wherein the interactions and miscibility of components in cubosomes spread at the interface are favorable compared to those in the monolayer generated from the sample of individual components mixed in chloroform. It is confirmed by the values of the thermodynamic functions obtained in the analysis of the compression–decompression hysteresis. The important structuring role of the Pluronic F108 polymer in the cubosome system reported earlier by Valldeperas et al. based on neutron reflectometry is also seen in our approach.^34^ Strong compression of cubosomal layers (beyond the limits of physiological pressure) leads to the removal of the polymer stabilizer from the monolayer assembly, and the characteristics of the cubosome nanostructures are lost.

Cubosome-derived layers on a solid substrate (mica) were fabricated using two different methods: the LB–LS transfer from the air–water interface and adsorption directly from the solution. The topographies of the dry layers showed the complete disintegration of the original cubosomal structures and the formation of a nonhomogeneous film on the mica surface. Interestingly, the AFM images of the films obtained by the combined Langmuir–Blodgett and Langmuir–Schaefer approach taken in water instead of air indicated the presence of some cubosomes on the solid surface. It suggests that cubosomes diffusing to the monolayer-covered air–water interface during the Langmuir–Schaefer (horizontal touch) transfer step may preserve their intact cubosomal form. This made us use the direct adsorption method from the bulk solution, which confirmed the presence of numerous characteristic cubosome structures on the mica surface. Since cubosomes are prepared by hydrating the GMO in the presence of water and a Pluronic F108 stabilizer, they require an aqueous environment to maintain their structure at the interfaces, which is also how they function in biological systems.^34,35^ Therefore, it has to be emphasized that in order to maintain the stability of cubosomes, preserving aqueous environment is essential.^35−37^ These conditions would be also necessary when the cubic nanoparticles are investigated as drug delivery vehicles, e.g., in monitoring drug release mechanisms at the interfaces. Our approach combining L-B and L-S techniques with AFM provides an explanation in the ongoing discussion of what happens to lipid nanoparticles with or without the cargo when they come into contact with an interface.

## Supporting Information Available

Additional atomic force microscopy images showing the topography of GMO, Pluronic F108 layers, and topography of bigger pattern of GMO/Pluronic F108, cubosome-derived layers, and layers obtained by spreading cubosome solution (PDF)
