# Supplementary material for: Interfacial Behavior of Cubosomes: Combined Langmuir–Blodgett/Langmuir–Schaefer and AFM Investigations
Source: Langmuir. 2023 May 26;39(22):7958–67. doi: 10.1021/acs.langmuir.3c00810 (PMC10249408; doi:10.1021/acs.langmuir.3c00810)
Supplement: Supplementary file 1 — la3c00810_si_001.pdf [file la3c00810_si_001.pdf]

Supporting Information

Interfacial Behavior of Cubosomes – Combined Langmuir–Blodgett/Langmuir–Schaefer and AFM Investigations

Michalina Zaborowska[1], Aleksandra Bartkowiak[1], Ewa Nazaruk[1], Dorota Matyszewska[2], Renata Bilewicz[1,2]

[1]*Faculty of Chemistry, University of Warsaw, Pasteura 1, 02093 Warsaw, Poland*

[2]*Faculty of Chemistry, Biological and Chemical Research Centre, University of Warsaw, Żwirki i Wigury 101, 02089 Warsaw, Poland*

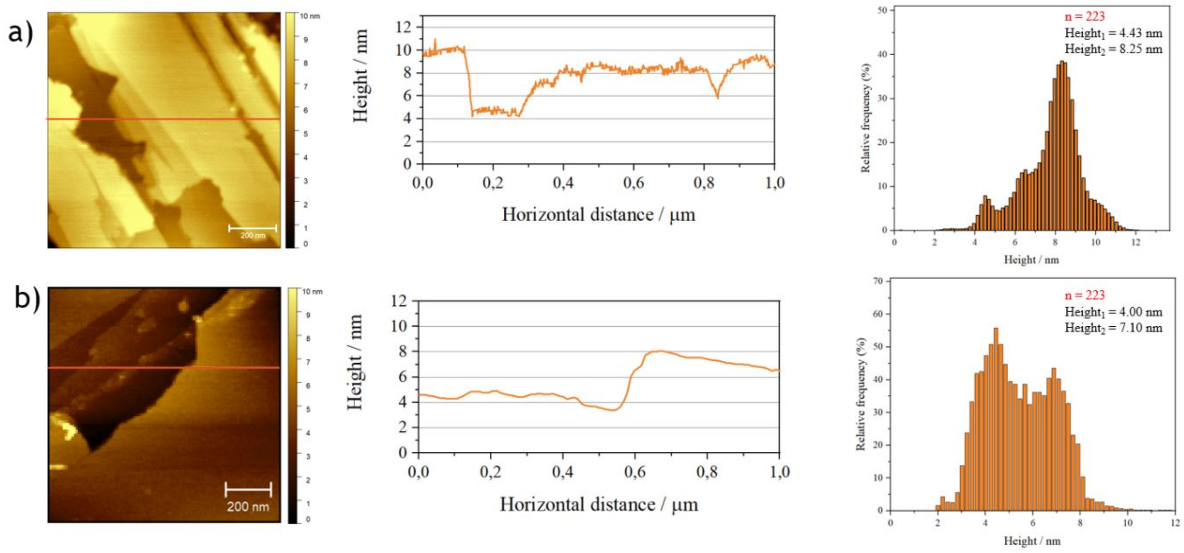

Figure S1 Topographic AFM images of GMO layers obtained by LB-LS method at surface pressure of 20 mN/m a) in air; b) in water (left column). The orange line corresponds to profile (middle column). Average height distribution histogram collected in the area of the given image (right column).

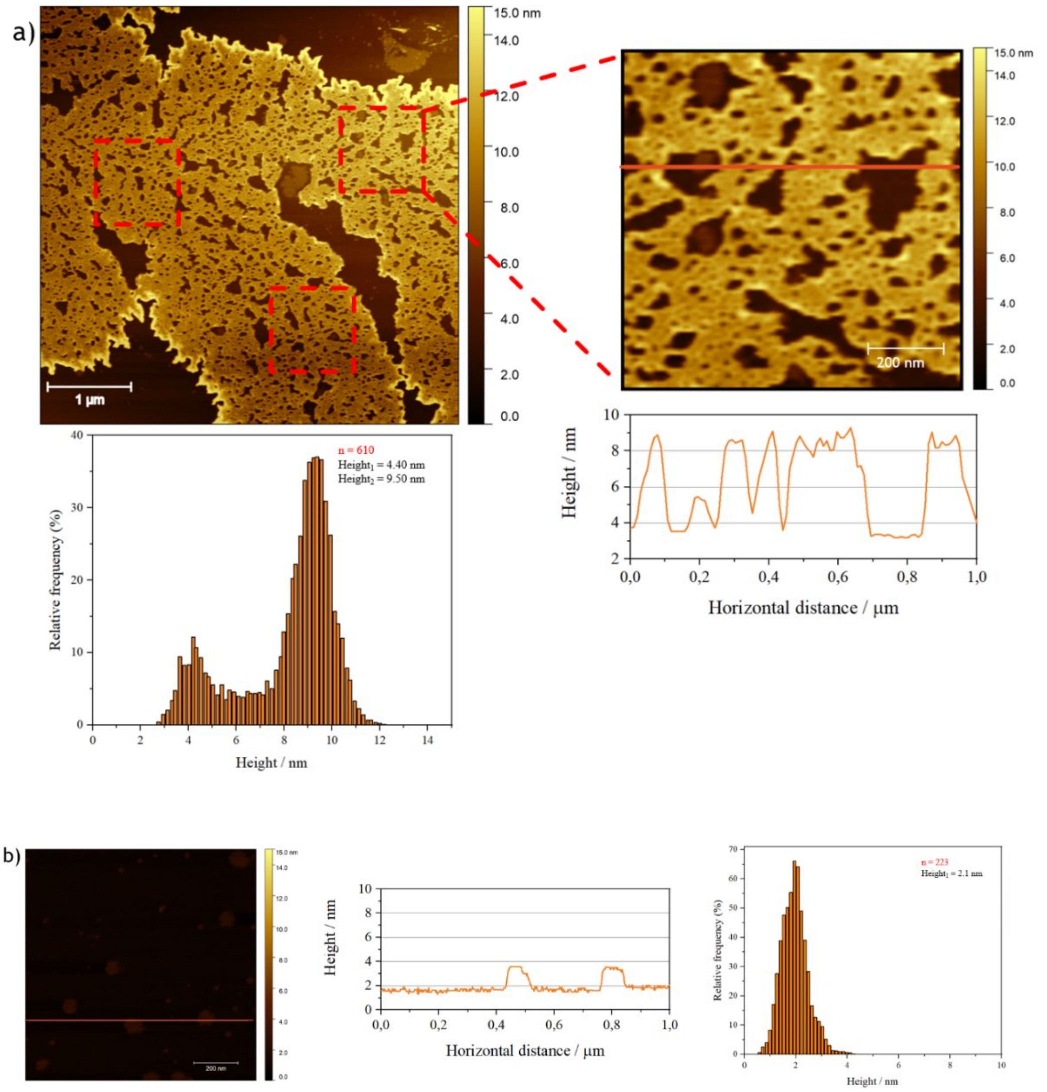

Figure S2 Topographic AFM images of polymer Pluronic® F108 layers obtained by LB-LS method at surface pressure equal to 20 mN/m a) in air; b) in water. The orange line correspond to profile. Average height distribution histograms collected in the selected areas (red dashed squares) of a given image.

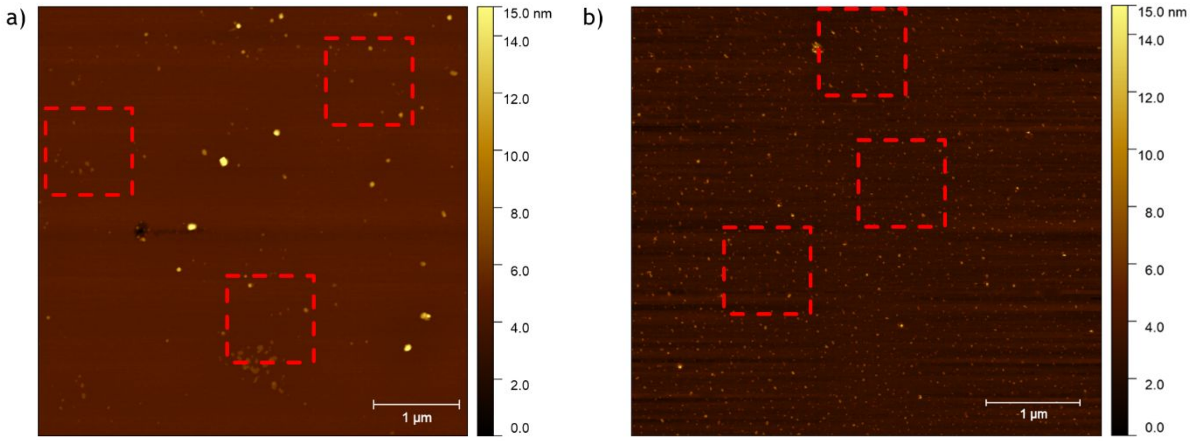

Figure S3 Topographic AFM images of the mixed GMO/Pluronic-F108 layers obtained by LB-LS method at surface pressure 20 mN/m a) in air; b) in water. Patterns size 5 x 5 μm.

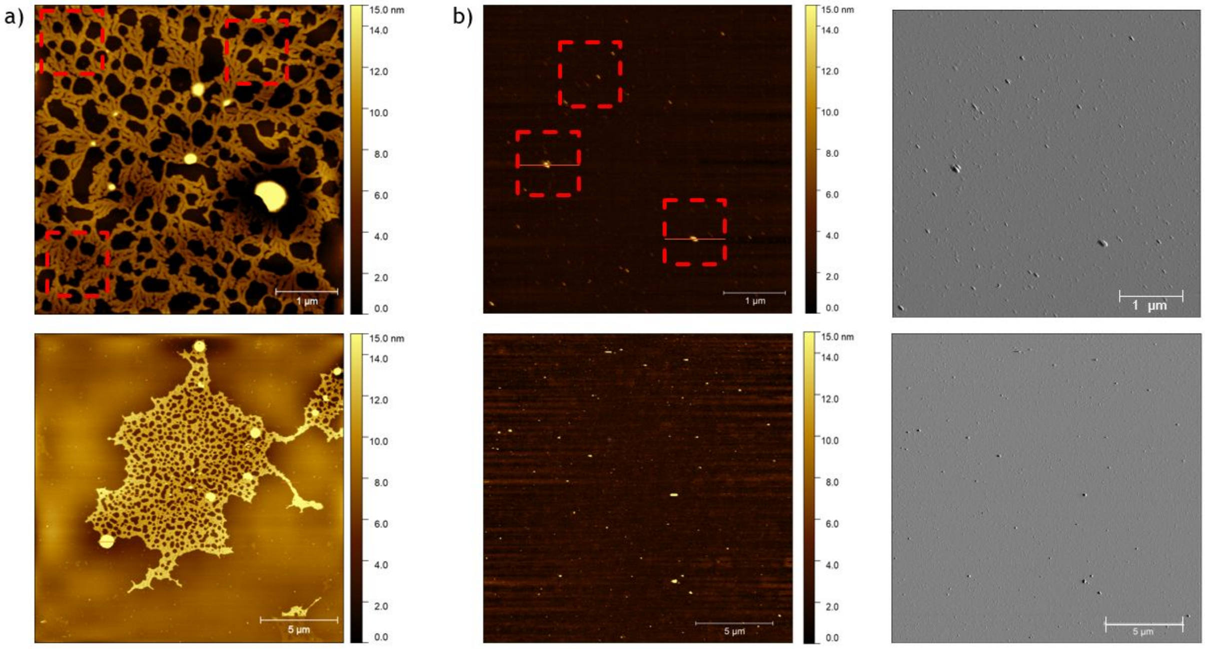

Figure S4 Topographic AFM images of the cubosome-derived layers obtained by LB-LS method at surface pressure of 20 mN/m measured a) in air; b) in water.

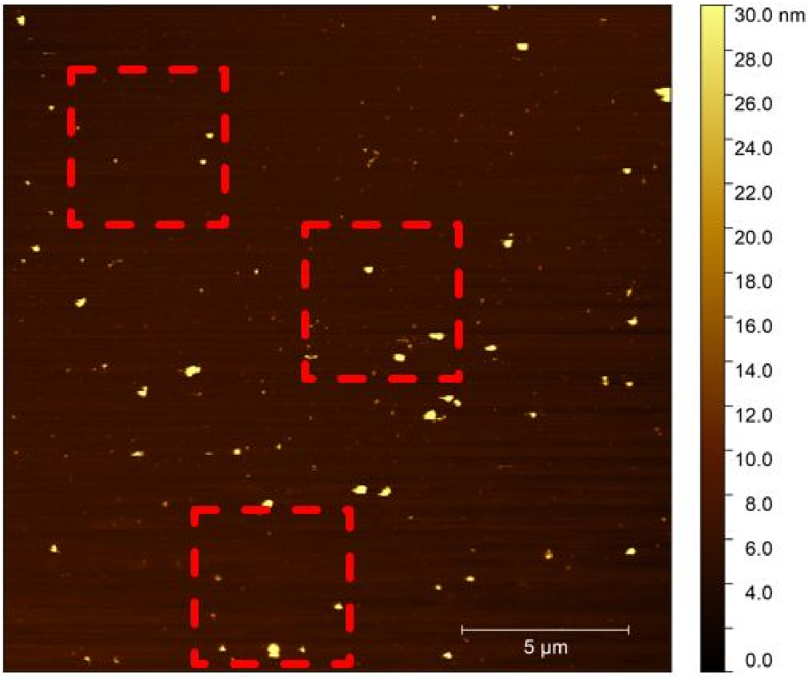

Figure S5 Topographic AFM images of the cubosomes layers obtained by spreading cubosome solution on the mica surface for adsorption time - 4 h and measured in water.
